# Generic atorvastatin is as effective as the brand-name drug (LIPITOR^®^) in lowering cholesterol levels: a cross-sectional retrospective cohort study

**DOI:** 10.1186/s13104-017-2617-6

**Published:** 2017-07-17

**Authors:** Alexander Loch, Jan Philipp Bewersdorf, Daniel Kofink, Dzafir Ismail, Imran Zainal Abidin, Ramesh Singh Veriah

**Affiliations:** 1 Department of Medicine, University Malaya Medical Centre, Kuala Lumpur, Malaysia; 2 Laboratory of Experimental Cardiology, Department of Cardiology, Division of Heart and Lungs, University Medical Center Utrecht, Utrecht, The Netherlands

**Keywords:** Generic medication, Lipid lowering drugs, Atorvastatin, Lipitor^®^, Hypercholesterolaemia, Efficacy

## Abstract

**Background:**

In a world of ever increasing health care costs, generic drugs represent a major opportunity to ensure access to essential medicines for people who otherwise would be unable to afford them. However, some clinicians and patients are still questioning the safety and effectiveness of generic formulations compared to the proprietary drugs necessitating further systematic research analyzing the generic drugs’ efficacy. Our objective was to compare the lipid lowering effects of generic and branded atorvastatin.

**Methods:**

This cross-sectional, retrospective cohort study was conducted at the University of Malaya Medical Centre from 1 May 2013 until 30 May 2013. We analyzed the lipid profiles (total cholesterol, LDL-cholesterol, HDL-cholesterol, triglycerides) of 629 patients before and at least 3 months after switching them from proprietary atorvastatin (Lipitor^®^) to generic atorvastatin (atorvastatin calcium from Ranbaxy Laboratories, Inc.). We also investigated if there was any difference in the effectiveness of both atorvastatin formulations in various ethnic groups.

**Results:**

266 patients were included in this study. When comparing the median values we found no statistically significant differences (Wilcoxon signed-rank test; p < 0.05) between proprietary and generic atorvastatin in lowering total cholesterol (4.60 mmol/l pre-transition vs. 4.50 mmol/l post-transition; p = 0.583), LDL-cholesterol (2.42 mmol/l vs. 2.41 mmol/l; p = 0.923) and triglycerides (1.50 mmol/l vs. 1.50 mmol/l; p = 0.513). While there was a statistically significant (p = 0.009) difference in HDL-cholesterol levels favouring proprietary atorvastatin, the extent of this change (1.26 mmol/l vs. 1.25 mmol/l) was deemed not to be clinically relevant. There was no statistically significant difference when analyzing the effects on various ethnic groups.

**Conclusions:**

Substituting proprietary atorvastatin for its generic formulation atorvastatin calcium does not result in a less effective management of hyperlipidemia. Our findings lend support to the approach of lowering health care costs by switching patients from branded drugs to their less expensive generic analogues.

## Background

In a world of ever increasing health care costs, generic drugs represent a major opportunity to cut health care costs and to ensure access to essential medicines for people who otherwise would be unable to afford them.

A generic drug must contain the same active ingredients and must be identical or within an acceptable bioequivalent range to the brand-name counterpart with respect to pharmacokinetic and pharmacodynamic properties. Generic manufacturers solely develop bioequivalent versions to existing drugs without having to prove the safety and efficacy of the drugs through clinical trials. In the United States the Drug Price Competition and Patent Term Restoration Act, also informally known as the Hatch–Waxman Act, states that pre-clinical and clinical testing does not have to be repeated for generics. A generic drug is considered to be bioequivalent to the brand name drug if the rate and extent of absorption do not show a significant difference from the listed drug [1].

There is however a perception among some patients and physicians that generics are inferior drugs. Patients are accustomed to their branded drugs and are often unwilling to change, particularly in the face of company sponsored advertising negating the benefits of generic drugs. Physicians commonly have negative perceptions of generic drugs, attitudes created and cemented by company marketing and information policies [2].

Atorvastatin, a lipid lowering agent marketed under the trade name Lipitor^®^ by Pfizer Inc. entered the market in 1996 and became the world’s best-selling drug of all time. Pfizer’s patent on atorvastatin expired in November 2011. Other manufacturers began to supply the generic versions of the drug by May 2012. The first company to develop a generic atorvastatin (known as atorvastatin calcium) and to introduce it to the market was Ranbaxy Laboratories, India’s largest pharmaceutical company [3]. Both the patient’s and the physician’s perception of Ranbaxy’s generic atorvastatin have been shaken by various quality control issues resulting in recalls and fines [4, 5]. Negative perceptions based on quality control issues invariably will result in negative assumptions about the therapeutic efficacy of generic drugs as well.

It is difficult to dispel negative perceptions regarding generic atorvastatin in general, as there are only very few studies available analysing the efficacy of non-proprietary atorvastatin [6, 7]. The data interpretation of these studies is limited for various reasons: low number of study subjects, the absence of reference groups and the fact that a multitude of generic and proprietary statins were examined.

The objective of our study was to analyse the lipid lowering effects of generic atorvastatin calcium (Ranbaxy) compared with the original brand-name drug (Lipitor^®^) in a real life population.

## Methods

### Study design

**Fig. 1:**
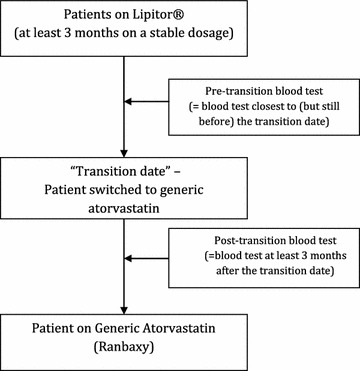
Study flow chart

This cross-sectional retrospective study was conducted at the University of Malaya Medical Centre from 1 May 2013 until 30 May 2013. The hospital pharmacy started dispensing generic, non-proprietary atorvastatin (Ranbaxy Laboratories) on 3 July 2012 as part of a general policy to switch all drugs to generics if possible in order to reduce costs. Prior to this, all patients with a prescription for atorvastatin were issued Lipitor^®^. All patients switched from proprietary to generic atorvastatin were identified from the pharmacy’s electronic drug prescription system. Lipid levels before and after the transition were compared. Figure 1 outlines the study flow chart.

### Data collection

The Medical Ethics Committee at the University Malaya Medical Centre approved the study and waived the necessity for informed consent to be obtained from each patient included (MEC ID No. 20152-1019). The data collected for this study included patient demographics, date of onset of branded atorvastatin therapy, date of first issuance of generic atorvastatin and adjunctive lipid-lowering medications. The date of the first issuance of the generic atorvastatin was considered the “transition date”. Low density lipoprotein (LDL)-cholesterol, total cholesterol, high density lipoprotein (HDL)-cholesterol and triglyceride levels were extracted from the electronic blood investigation reporting system for the time before and after the transition. When multiple blood tests existed for a patient, the blood test closest to the transition date, but not later than the transition date was analysed as the pre-transition test. Post-transition blood testing had to be at least 3 months after the transition date. For patients with multiple blood tests following the transition, the first blood test after the 3 month period after switching was utilised for data analysis.

### Inclusion and exclusion criteria

All patients with a record of transition from proprietary to generic atorvastatin were included. Patients had to be on atorvastatin therapy for at least 3 months to be included. Exclusion criteria were as follows: patients with missing pre- or post-transition blood results, patients who were started on concurrent lipid-modifying medications during the transition period and patients with atorvastatin dose changes during the transition period.

### Study outcomes

This study assessed the differences between pre-transition and post-transition lipid levels.

### Statistical methods

Differences between pre- and post-transition lipid levels were tested for normality by Shapiro–Wilk tests (p < 0.05) and were found to significantly deviate from normality. Changes in lipid levels were therefore analysed with the use of the non-parametric Wilcoxon signed-rank test. Two-sided p values <0.05 were considered statistically significant. Statistical analyses were performed in R (version 3.2.4).

## Results

### Study population

A total of 629 patients were switched from Lipitor^®^ to the generic drug. 363 patients had to be excluded from analysis for various reasons (missing lipid values pre- and post-transition, dose changes between pre-and post-transition or introduction of other lipid lowering medications). The mean age of the study population was 64.3 (±9.5) years. Male and female patients were equally represented. Patients were divided according to ethnicity to allow analysis for inter-ethnic differences. 63 patients were on lipid-lowering medications other than atorvastatin. These were exclusively fenofibrate and ezetimibe. The dose of the lipid-lowering medication was stable throughout the study period. Pre-transition blood tests were done 99.5 days (median) before the transition date. Post-transition blood tests were done 180 days (median) after the transition date. A description of the study population is shown in Table 1.

**Table 1:** Demographic details of the study population

Study population	N = 629
Patients excluded (due to missing blood tests, dose changes)	N = 363
Patients included	N = 266
Mean age	64.3 (±9.5)
Racial setup	
Malay	N = 126
Chinese	N = 71
Indian	N = 69
Gender	
Male	N = 136
Female	N = 130
Patients on lipid lowering drugs other than atorvastatin	N = 63
Time pre-transition blood test until transition date	Median 99.5 days
	Mean 123.3 days
Time transition date until post-transition blood test	Median 180 days
	Mean 209 days

### Lipid levels pre and post transition

The median triglyceride pre-transition value was 1.50 mmol/l (mean 1.71 mmol/l; range 0.40–7.00 mmol/l) and the post-transition value was 1.50 mmol/l (mean 1.72 mmol/l; range 0.40–7.70 mmol/l) with p = 0.513. The median LDL pre-transition value was 2.42 mmol/l (mean 2.64 mmol/l; range 0.47–6.34 mmol/l) and the post-transition value was 2.41 mmol/l (mean 2.64 mmol/l; range 1.01–7.30 mmol/l) with p = 0.923. The median HDL pre-transition value was 1.26 mmol/l (mean 1.29 mmol/l; range 0.12–2.51 mmol/l) and the post-transition value was 1.25 mmol/l (mean 1.26; mmol/l range 0.15–2.39 mmol/l) with p = 0.009. The median total cholesterol pre-transition value was 4.60 mmol/l (mean 4.71 mmol/l; range 1.76–8.40 mmol/l) and the post-transition value was 4.50 mmol/l (mean 4.68 mmol/l; range 2.30–9.90 mmol/l) with p = 0.583.

There were no statistical differences between pre- and post-transition levels (Wilcoxon signed-rank test) for triglycerides, LDL-cholesterol, and total cholesterol. The statistical analysis for HDL-cholesterol yielded a significant p value, however in practical terms, it is obvious that a mean HDL of 1.29 mmol/l pre-transition vs. 1.26 mmol/l post-transition is of little clinical relevance.

**Fig. 2:**
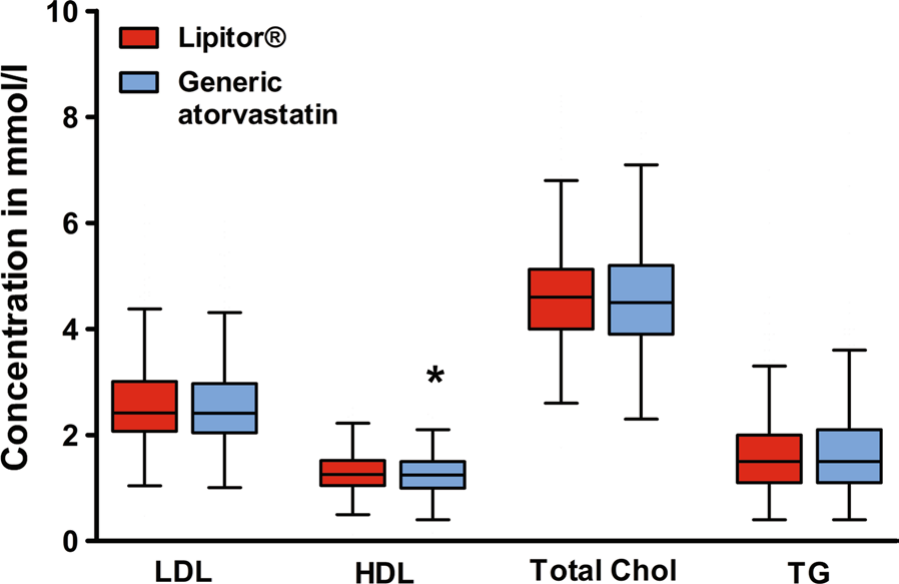
Box- and whisker plot of mean blood concentrations of LDL-cholesterol (LDL), HDL-cholesterol (HDL), total cholesterol (total chol) and triglycerides (TG) with boxes indicating the upper and lower quartiles. Tukey method was used to plot whiskers. Outliers are not shown in the diagram. *Asterisk* represents a statistically significant difference in HDL-cholesterol levels before and after the transition (p < 0.05; Wilcoxon signed-rank test)

There were no differences when analysed according to ethnicity. Figure 2 and Table 2 visualize the presented data.

**Table 2:** Lipid values before and after transition from branded to generic atorvastatin

	Pre-transition lipid values (mmol/l)	Post-transition lipid values (mmol/l)	Difference of mean (Wilcoxon signed-rank test)
TG	Median 1.50	Median 1.50	V = 14304, p = 0.513
	Mean 1.71	Mean 1.72	
	Range 0.40–7.00	Range 0.40–7.70	
LDL	Median 2.42	Median 2.41	V = 16333, p = 0.923
	Mean 2.64	Mean 2.64	
	Range 0.47–6.34	Range 1.01–7.30	
HDL	Median 1.26	Median 1.25	V = 14039, p = 0.009
	Mean 1.29	Mean 1.26	
	Range 0.12–2.51	Range 0.15–2.39	
Total cholesterol	Median 4.60	Median 4.50	V = 15058, p = 0.583
	Mean 4.71	Mean 4.68	
	Range 1.76–8.40	Range 2.3–9.9	

## Discussion

We could demonstrate in this cross-sectional cohort study, that the lipid lowering properties of generic atorvastatin calcium (Ranbaxy Laboratories, Inc.) are comparable to those of Lipitor^®^ (Pfizer, Inc.). Negative perceptions about generic atorvastatin with regards to efficacy appear to be unfounded.

Our findings are in keeping with other studies comparing branded and generic atorvastatin. A retrospective study by Rahalkar et al. showed no differences in plasma levels of total or LDL-cholesterol, or triglycerides, but was associated with a small but significant increase in HDL-cholesterol [7]. Kim et al. studied 211 Korean patients for the purpose of marketing a Korean generic formulation and demonstrated equal efficacy and tolerability of generic and proprietary atorvastatin [8]. Ong et al. reported good LDL-cholesterol reduction with generic atorvastatin in 85 Malaysian patients without serious drug related adverse events, however without having a control group [9].

Generic drugs are the only option for millions of people to obtain affordable pharmacotherapy, particularly in developing countries. The fact that generic manufacturers do not have to prove the safety and efficacy of the drugs through clinical trials makes it of utmost importance for the manufacturers to uphold highest production standards to avoid loss of confidence in their products and to maintain patient safety. Reports of impurities, contaminations, dosing errors and misrepresentation of generic drug data do not mean that generic substitutions are not equivalent or ineffective. But these reports highlight the importance of regulatory oversight from the US Food and Drugs Administration and other national bodies [4, 5].

There have been numerous studies demonstrating comparable levels of effectiveness and safety for various generic drugs [10–12]. Despite this growing level of evidence, the perception that generic drugs are inferior remains among some patients and physicians. Patients are accustomed to their branded drugs and are often anxious about having to change to generic formulations [13]. Physicians and pharmacists commonly have negative perceptions of generic drugs with one study showing that about a third of pharmacists considers generic drugs less effective than their branded analogues [2, 14]. We could demonstrate with our study that negative perceptions with regards to the efficacy of generic atorvastatin seem to be unfounded.

This is a retrospective cohort study with all its associated limitations. Data on drug compliance was not available. However, our hospital pharmacy dispenses a maximum supply of 2 months of medication only. Non-compliance would be detectable by patients not collecting the renewal prescription after that 2 month period. Data were collected by chart abstraction which always poses the possibility for mistakes. It is of note, that only Lipitor^®^ from Pfizer, Inc. and atorvastatin calcium from Ranbaxy Laboratories, Inc. were investigated. Therefore, the study conclusions might not be fully transferable to other generic atorvastatin formulations. Our study was a cross-sectional retrospective cohort study. We did not attempt to analyse lipid profiles over time. It would have been ideal to follow the patients prospectively over a longer period of time to assess the long-term efficacy of generic atorvastatin in the management of hyperlipidemia. Further studies are warranted to address these questions.

## Conclusions

Substituting proprietary atorvastatin for its generic formulation atorvastatin calcium does not result in a less effective management of hyperlipidemia. Our findings lend support to the approach of lowering health care costs by switching patients from branded drugs to their less expensive generic analogues. Further studies in the field of generic medication efficacy are desirable to supply physicians with the data and confidence to prescribe these medications.

## Abbreviations

Chol: cholesterol
d: days
HDL: high-density lipoprotein
LDL: low-density lipoprotein
TG: triglycerides
